# The effect of intermittent preventive treatment on anti-malarial drug resistance spread in areas with population movement

**DOI:** 10.1186/1475-2875-13-428

**Published:** 2014-11-15

**Authors:** Miranda I Teboh-Ewungkem, Jemal Mohammed-Awel, Frederick N Baliraine, Scott M Duke-Sylvester

**Affiliations:** Department of Mathematics, Lehigh University, Bethlehem, PA 18015 USA; Department of Mathematics, Valdosta State University, Valdosta, GA 31698 USA; Department of Biology, LeTourneau University, Longview, TX 75602 USA; Department of Biology, University of Louisiana at Lafayette, Lafayette, LA 70504 USA

**Keywords:** Malaria parasite, Drug resistance, Malaria control, Low transmission area, High transmission area, Interacting regions, IPT

## Abstract

**Background:**

The use of intermittent preventive treatment in pregnant women (IPTp), children (IPTc) and infant (IPTi) is an increasingly popular preventive strategy aimed at reducing malaria risk in these vulnerable groups. Studies to understand how this preventive intervention can affect the spread of anti-malarial drug resistance are important especially when there is human movement between neighbouring low and high transmission areas. Because the same drug is sometimes utilized for IPTi and for symptomatic malaria treatment, distinguishing their individual roles on accelerating the spread of drug resistant malaria, with or without human movement, may be difficult to isolate experimentally or by analysing data. A theoretical framework, as presented here, is thus relevant as the role of IPTi on accelerating the spread of drug resistance can be isolated in individual populations and when the populations are interconnected and interact.

**Methods:**

A previously published model is expanded to include human movement between neighbouring high and low transmission areas, with focus placed on the malaria parasites. Parasite fitness functions, determined by how many humans the parasites can infect, are used to investigate how fast resistance can spread within the neighbouring communities linked by movement, when the populations are at endemic equilibrium.

**Results:**

Model simulations indicate that population movement results in resistance spreading fastest in high transmission areas, and the more complete the anti-malarial resistance the faster the resistant parasite will tend to spread through a population. Moreover, the demography of infection in low transmission areas tends to change to reflect the demography of high transmission areas. Additionally, when regions are strongly connected the rate of spread of partially resistant parasites (R1) relative to drug sensitive parasites (RS), and fully resistant parasites (R2) relative to partially resistant parasites (R1) tend to behave the same in both populations, as should be expected.

**Conclusions:**

In fighting anti-malarial drug resistance, different drug resistance monitoring and management policies are needed when the area in question is an isolated high or low transmission area, or when it is close and interacting with a neighbouring high or low transmission area, with human movement between them.

**Electronic supplementary material:**

The online version of this article (doi:10.1186/1475-2875-13-428) contains supplementary material, which is available to authorized users.

## Background

Malaria is a vector-borne disease caused by members of the genus *Plasmodium* that continue to afflict many countries. An estimated 3.4 billion people are at risk of the disease, of whom about 35.3% are at higher risk [1]. In 2012, an estimated 207 million malaria cases occurred resulting in an estimated 627,000 deaths, with about 77% of the deaths being of children under five years of age [1]. Efforts to control the disease are ongoing and continue to evolve as the challenges evolve. New control strategies and approaches have been proposed to tackle new challenges with the short term goal of disrupting disease transmission and reducing disease burden, and a long term goal of reducing disease impact on populations and potentially achieving disease eradication. Intermittent Preventive Treatment (IPT) is a malaria control strategy aimed at reducing disease burden and parasite load in the most vulnerable segments of the population.

In malariology, intermittent preventive treatment (IPT) denotes the administration of a full curative course of an anti-malarial drug at particular time points to the most malaria-vulnerable sub-population, regardless of their malaria infection status. The sub-populations to which IPT is typically applied are defined by specific at-risk groups, including infants, in which case it is referred to as IPTi, children (IPTc) and pregnant women (IPTp). This increasingly popular preventive strategy aims at diminishing the incidence of clinical malaria in these vulnerable groups. Thus, IPT helps minimize deaths and adverse pregnancy outcomes such as maternal anaemia, intrauterine growth retardation, abortion, or low birth weights, while allowing for the normal development of natural immunity in children [2]. The downside of IPT is that once drug concentrations fall below therapeutic levels there is a risk of reinfection and selection for drug resistance [3, 4].

Historical evidence suggests that anti-malarial drug resistance tends to develop from areas of low malaria transmission [5–7]. In particular, available data suggest that resistance to both chloroquine (CQ) and sulphadoxine-pyrimethamine (SP) emerged from areas of low or unstable transmission. Low transmission areas (within highly endemic regions) are typically located in upland sites where temperatures tend to be cooler because of the high altitude, and rainfall accumulation tend to be smaller [8–10]. These conditions make it less conducive for mosquitoes to reproduce, hence lowering malaria transmission. On the other hand, high transmission areas are typically found at lower elevations [10] where the temperatures are relatively warmer, there is less run-off, and hence more standing water. These conditions are more conducive for mosquito breeding and hence reproduction, and malaria transmission.

Hypotheses proposed to explain why CQ-resistance originated from the presumed low or unstable transmission areas outside of sub-Saharan Africa, include: (i) a lower frequency of resistant alleles in higher transmission areas because of within-host competition [5, 11]; (ii) less drug treatment (per parasite) in higher transmission areas [5, 6, 11]; and (iii) mutant parasites are less likely to survive a host immune response in high transmission areas, where immunity is better developed [5, 12]. Quantitative population genetic and epidemiological models have also offered an explanation for why resistance is less likely to emerge in high transmission areas [5, 13]. Higher transmission intensity is associated with a higher level of clinical immunity to malaria, due to frequent exposure of residents to the parasites [5]. The term “clinical immunity” refers to the gradually acquired, parasite-exposure-primed immune response that enables individuals to be symptom-free even when they have the parasites in their blood [14]. Consequently, the rates of anti-malarial drug use are lower among clinically immune individuals, since they rarely feel sick from the infection [5]. The relatively lower usage of anti-malarial drugs by clinically immune individuals undercuts the selective advantage of resistant parasites and creates a natural refuge for the drug-sensitive parasites, making it more conducive for sensitive parasites to thrive. The model in [5] demonstrates that the existence of a refuge for drug sensitive parasites in high-transmission areas can slow or prevent the evolution of anti-malarial drug resistance.

Once anti-malarial drug resistance has emerged in a particular area, the factors that influence its rate of spread remain unclear [10]. Moreover, the issue of whether drug resistance spreads faster in low or high malaria transmission areas remains controversial [10]. In particular, some predictions suggest that resistance may spread faster in areas of low transmission [10, 15, 16], while others have predicted faster spread in areas of high transmission [10, 17]. Other hypotheses predict that only partially resistant parasites spread faster in areas of low transmission while only fully resistant ones spread faster in areas of high transmission [5, 18]. These inconsistencies may be due to the multiplicity of factors involved in the evolution of drug resistance and spread of malaria parasites, including climatic factors, geographical factors, human host factors and parasite and vector factors, as well as the assumptions made in modelling malaria transmission and drug resistance.

Available models of malaria drug resistance that include IPT predict rapid spread of partially drug resistant malaria strains in areas of low transmission, and rapid spread of fully resistant strains in areas of high malaria transmission [18]. However, none of the currently available models explicitly consider the effects of spatial structure of the population. Spatial structure is important, particularly in places like Africa where there exists many situations where low and high malaria transmission areas are in close proximity to each other, with continuous human movement between the two epidemiological settings [19]. It has been shown that human movement from the low transmission highland regions to the high transmission lowland regions increases the risk of malaria infections in the low transmission highland regions [20, 21]. Hence, there is reason to believe that human movement between transmission settings plays a role in shaping parasite population structure [20, 21], and thus affects the rate of spread of anti-malarial drug resistance.

In view of the above findings, it is critical to understand how implementation of IPT in differing but connected transmission areas may affect the spread of drug-resistant parasites. The present model adds spatial structure to a previously published non-spatial/non-movement model developed in [18] to investigate the impact of human movement between high and low transmission areas on the spread of drug-resistant malaria when infants undergo IPT (i.e. IPTi). The spatial model predicts that for movement rates in which humans stay for hours or days up to about 14 years, resistance (both partial and full) always spreads faster in high transmission areas and that the more complete the resistance the faster the resistant parasites spread through a population. These results are not a function of the dosage regimen used in IPTi. Thus, the prediction by the spatial model differs from the prediction by the non-spatial model [18], which suggested that the rate of spread of resistant parasites in low or high transmission settings depends on the degree of resistance. Taken together, the predictions based on the spatial/movement versus the non-spatial/non-movement model suggest different public health policies for monitoring the rate of spread of anti-malarial drug resistance in different malaria transmission settings under IPTi.

## Methods

A system of ordinary differential equations (ODEs) is used to model the spread of drug-resistant malaria parasites within interacting human populations. Focus is placed on the malaria causing agent, the *Plasmodium* parasites, and how they spread in a human population. The ecology of *Anopheles* mosquitoes, the agents responsible for transmitting the parasites from one human to the other, is implicitly handled in the transmission rate between human hosts. Derivation of the model equations follows the approach used in [18], with spatial structure added in the form of two linked interacting populations with movement from one to the other. The populations may differ in size, and differ in the rate at which their respective members experience transmission (either high or low). The effect of differences in the relative sizes of the linked populations on the spread of resistance are examined, as well as the potential for asymmetric movement between the populations. The model tracks humans receiving anti-malarial drugs as prophylactic treatment via IPTi or for the treatment of symptomatic infections. The model also identifies potential human hosts available for infection by a sensitive or resistant parasite. Three levels of parasite resistances are included: (i) parasites that possess no resistance to anti-malarial drugs (RS), (ii) parasites that possess an intermediate degree of resistance (R1), and (iii) refractory parasites (R2).

In the model, human hosts are divided into categories based on their infection status and whether or not they are receiving treatment for symptomatic infections, or via IPTi. Categories are proportions of susceptible humans, infected humans, symptomatic-treated humans, humans receiving IPT, or temporarily immune humans. The chemoprophylaxis and IPTi drugs considered are sulphadoxine-pyrimethamine (SP), a drug with a long half-life (148-256 hours), and chlorproguanil (CPG)-dapsone (DDS) (CPG-DDS), a drug with a short half-life (27-35 hours) [22, 23]. Drugs with long half-lives are slowly eliminated from the body compared to those with shorter half-lives, and are therefore expected to impose greater selective pressure for drug resistance than those with shorter half-lives [24]. The expectation is that drugs that persist longer in the body at sub-therapeutic levels will provide more opportunities for non-resistant (susceptible) parasites to acquire resistant traits, and for partially resistant parasites to become fully resistant. Infection by fully resistant parasites is assumed to be untreatable.

The fitness of each of the three classes of parasites (susceptible, partially resistant and fully resistant) is assessed as the fraction of the human population that can be infected by each parasite class at the endemic equilibrium, taking into consideration the average duration of infection. For example, fully drug-susceptible and partially drug-resistant parasites can infect hosts receiving no treatment. However, partially resistant parasites have higher relative fitness than the fully susceptible ones because they can also infect hosts that are being treated, when the concentration of the drug drops to sub-therapeutic levels. The rate at which resistance spreads increases as the relative fitness of the parasites increases.

### The human dosing model with movement

**Figure 1:**
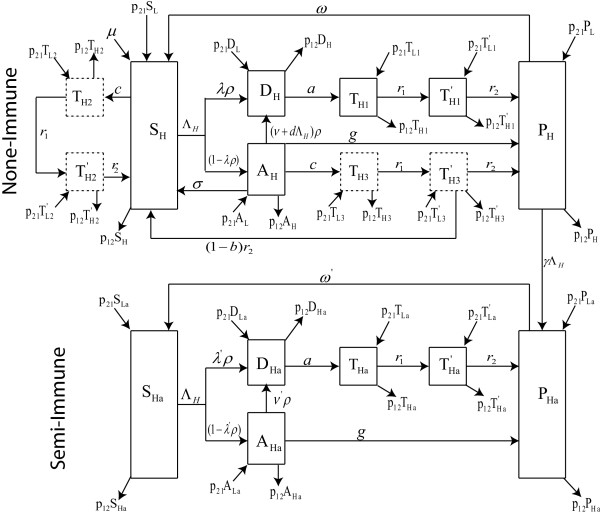
**Model schematic.** Schematic diagram of the movement model for the high transmission area showing disease progression in non-immune and semi-immune individuals. The schematic of the movement in the low transmission area is similar to that of the high transmission area ith the following changes: all variables indexed *H* become variables indexed *L* and vice versa, while all movements parameters *p*_*ij*_ become *p*_*ji*_ parameters in the low transmission case.

The processes that govern the spread of infection are illustrated in Figure 1 and are governed by equations (1H) and (1L) [see Additional file 1]. All individuals are born as susceptible non-immune *S*_*i*_ at a constant birth rate *μ*, where *i* indexes the population as either in a high (*i* = *H*) or low (*i* = *L*) transmission setting. The model assumes that each population has reached a birth/death equilibrium and that the birth rate, *μ*, equals the mortality rate from all causes. Susceptible individuals (*S*_*H*_*,S*_*Ha*_) become infected at a rate Λ_*i*_ (the entomological inoculation rate (EIR) and subsequently experience a cycle of clearance which may be due to an immune response [25–27] or due to treatment. Treatment requires a dose of anti-malarial drug, which at therapeutic levels in an individual can clear any drug-sensitive parasites and prevent re-infection by partially resistant parasites. At sub-therapeutic levels, the drug concentration is at a level that is active against sensitive parasites but partially resistant and fully resistant parasites can still infect the individual. When drug concentrations fall to zero, individuals may develop temporal (short-lived) immunity [18, 27]. Repeated infection results in the development of longer-lived immunity [18, 28] in humans. Humans who have had repeated infection, mostly the adults in the population, are classified as semi-immune individuals *S*_*ia*_. Semi-immune individuals have a higher probability of carrying an asymptomatic infection due to longer continuous exposure to the malaria parasites [29].

Individuals receiving drug treatment are classified using the variable *T*_*x*_, where *x* identifies whether the treatment is administered to a non-immune or a semi-immune individual, and whether the treatment is administered because of symptomatic malaria infection or because of IPTi administration. If treatment is received because of a symptomatic malaria infection in semi-immune individuals, *x* = *a*, and in non-immune individuals, *x* = 1. If treatment is received because of IPTi administration, then *x* =2 when those receiving the IPTi are susceptible non-immune humans, and *x* = 3 when they are infected non-immune humans who have not yet sought treatment.

Upon infection with malaria parasites a fraction, *λ*, of individuals from the class of susceptible untreated non-immune individuals (*S*_*i*_), will exhibit symptoms, of which a fraction *ρ* will receive treatment and join the class of symptomatic infected and treated non-immune individuals (*D*_*i*_). The remaining fraction of infected individuals (1 -*ρλ*) do not receive immediate treatment either because their infections are not recognized or because of health care and financial limitations. These individuals move to the class of infected untreated non-immune individuals (*A*_*i*_).

Individuals in class *A*_*i*_ either clear their parasites slowly via immune mechanisms, achieve short-lived temporary immunity from reinfection at a rate *g* and join the class of uninfected non-immune individuals with temporary immunity, denoted by *P*_*i*_. Others clear their infection spontaneously without obtaining temporary immunity and return to the susceptible class *S*_*i*_ at rate *σ*. The remaining individuals in class *A*_*i*_ receive treatment and move into the treated class *D*_*i*_ either because they exhibit symptoms of their existing infection at a rate *ν*, or because they exhibit symptoms following a subsequent infectious bite, at a rate *d*Λ_*i*_. Collectively, these latter individuals move from *A*_*i*_ into *D*_*i*_ at a combined rate of (*ν* + *d*Λ_*i*_)*ρ*.

Treated non-immune individuals (*D*_*i*_) progress through three subsequent stages representing the stages during which drug concentrations are sufficient to clear parasite infection (*T*_*i*1_), drug levels are at sub-therapeutic levels (*T'*_*i*1_), and when drugs have been cleared from their system leading to the acquisition of temporary immunity to join class *P*_*i*_. The transition from *D*_*i*_ to *T*_*i*1_ occurs at rate *a* where 1*∕a* days is the mean time under treatment required for a drug to reach therapeutic levels. Drug concentrations decline in the body over an average period of 1*∕r*_1_ days, after which the *T*_*i*1_ individuals move to class *T'*_*i*1_. Individuals move from class *T'*_*i*1_ to class *P*_*i*_ at rate *r*_2_ where 1*∕r*_2_ days is the mean residence time for a drug to persist at sub-therapeutic levels. The time spent in the sub-therapeutic class, *T'*_*i*1_, is important because it is this period that provides a fitness advantage for partially and fully resistant parasites over the fully susceptible parasites [30].

Some individuals in the *P*_*i*_ class lose their temporary immune status at rate *w* so that 1*∕w* days later they join class *S*_*i*_. Here, 1*∕w* is the expected duration of immunity. Others, who have undergone repeated exposure, will transition to the class of semi-immune individuals with temporary immunity, denoted by *P*_*ia*_. This later transition occurs at the rate at which individuals become infected and exhibit symptoms (*γ*Λ_*i*_).

Individuals in class *S*_*ia*_, the class of uninfected and untreated semi-immune humans, can also be re-infected with the malaria parasites but their immune system is able to limit parasitaemia and they manifest symptoms with less severity. These individuals become infected at the same rate of Λ_*i*_ as non-immune individuals, but a smaller fraction of them *λ'*, exhibit symptoms and receive treatment with probability *ρ* to join the symptomatic infected and treated semi-immunes class *D*_*ia*_. The semi-immune individuals who do not immediately exhibit symptoms join the infected untreated semi-immunes class *A*_*ia*_. However, they may subsequently exhibit symptoms at rate *ν*^’^, receive treatment with probability *ρ* and join class *D*_*ia*_. Progression from class *D*_*ia*_ to class *P*_*ia*_ can occur without drug treatment as a result of an immune response that clears malaria parasites. This occurs at rate *g*, the same rate as in non-immune individuals. Individuals from class *D*_*ia*_ can also progress to class *P*_*ia*_ through different stages characterized by the concentration of drugs in their system: individuals with drug concentrations sufficient to clear infection (*T*_*ia*_); those with drug levels at sub-therapeutic levels (*T'*_*ia*_), and those who cleared the drugs from their system and have acquired temporary immunity (*P*_*ia*_). The transitions *D*_*ia*_ → *T*_*ia*_ → *T'*_*ia*_ → *P*_*ia*_ at the respective rates *a*, *r*_1_ and *r*_2_ are similar to that of the non-immune classes *D*_*i*_ → *T*_*i*1_ → *T'*_*i*1_ → *P*_*i*_, at the same respective rates. Humans in class *P*_*ia*_ lose their temporary immunity at a much slower rate of *w'* than the rate of *w* for non-immune individuals.

IPTi may be administered as chemoprophylaxis to non-immune individuals who are either susceptible (*S*_*i*_), infected and untreated (*A*_*i*_), or temporarily immune (*P*_*i*_). These individuals undergo IPTi drug dosing at a rate *c*. Here, IPTi treated susceptible non-immune humans (*S*_*i*_) will proceed through a similar stepwise process of drug concentrations with the transitions *S*_*i*_ → *T*_*i*2_ occurring at rate *c* as drugs reach therapeutic levels, *T*_*i*2_ → *T'*_*i*2_ at rate *r*_1_ as drug concentration goes from therapeutic to sub-therapeutic levels, and finally *T'*_*i*2_ → *P*_*ia*_ at rate *r*_2_, as drugs are purged. Similarly, infected but untreated non-immune humans *A*_*i*_ who receive a curative dose of IPTi for malaria follow the transition from class *A*_*i*_ → *T*_*i*3_ at rate *c* and *T*_*i*3_ → *T'*_*i*3_ at the same rate *r*_1_ as that for susceptible non-immune humans. However, only a fraction *b* of the individuals in class *T'*_*i*3_ develop temporary immunity to join the *P*_*i*_ class. The remainder, (1 - *b*), move directly to the susceptible class, with no acquired temporal protection. It is assumed that for the individuals in class *P*_*i*_ who receive IPTi, the loss or development of their immunity is not affected by the IPTi administration. In addition, if a non-immune individual arrives to receive an IPTi dose and is found to be clinically sick, the individual is treated and hence enter the treatment cycle through class *D*_*i*_.

In the present model formulation, the assumption is that the high and low transmission areas are close enough with continuous movement between them and that there is no change of disease status during the movement period. In the non-spatial model [18], the total populations in each transmission area (and hence both) were assumed to be a constant. Here, with the modification to include human movement between low and high transmission regions, that assumption remains unchanged. To illustrate this, and to understand the derivation of the additional movement terms in this model, the model variables are first considered in terms of total numbers and then appropriately scaled to proportions. An illustration for the *S*_*H*_ and *S*_*L*_ classes is provided; the rest will follow in a similar manner. Let 1*∕p*_12_ be the time (in days) a human resident in the high transmission area spends visiting the low transmission area, and 1*∕p*_21_ be the time (in days) a low transmission resident spends visiting the high transmission area. In addition, let *m* = *N*_*H*_*∕N*_*L*_ be the ratio of the total humans in the high transmission area (*N*_*H*_) to that of the low transmission area (*N*_*L*_), Λ_*H*_ be the EIR or infectious bites per person per day in the high transmission area and Λ_*L*_ be that in the low transmission area. Then the equations governing the susceptible untreated non-immune individuals for the high (*S*_*H*_) and low (*S*_*L*_) transmission areas are, respectively,


Without prior assumption that the total populations in the high and low transmission regions are constant, the equations governing the total human populations in the high (*N*_*H*_) and low (*N*_*L*_) transmission regions are


Upon rescaling to proportions,


so that the scaled *S*_*H*_ equation (in proportion) becomes


Simplifying yields the equation


where  constant. Note that the above analyses remain true under a constant population assumption in the high transmission region since, in that case, the term . A similar argument and calculation in the low-transmission region will yield


The analyses can be applied to the remaining equations to obtain the system of equations that govern the spread of infection for the human dosing model with movement in both the high and low transmission regions as equations (1H) and (1L), respectively [see Additional file 1]. The schematic representation is illustrated in Figure 1. Note that all equation variables are now in terms of proportions, and that for *p*_12_ = 0 = *p*_21_, the model in [18] is retrieved.

To understand the meaning of a constant total population in the model with movement, consider the term  and differentiate to obtain


So *m* = constant if and only if  if and only if *.* Therefore, for  precisely when *p*_*21*_*N*_*L*_ = *p*_*12*_*N*_*H*_*.* Alternatively, when  i.e. the total populations in each of the transmission areas (high and low) is constant. The equality *p*_*21*_*N*_*L*_ = *p*_*12*_*N*_*H*_, can be interpreted as follows: for a fixed average time, *t*, the total number of individuals moving from the low transmission area into the high transmission area at rate *p*_21_ is the same as that from the high transmission area to the low transmission area at rate *p*_12_. This can be thought of as a scenario in which the populations are at a dispersal equilibrium such that neither population is changing with the interaction. Since for  and an alternative interpretation is that the ratio of the movement rates is always proportional to the relative population sizes.

### The parasite fitness model and the spread of resistance

The spread of resistant parasites in the human dosing model described above is impacted by infection, treatment [3, 30–32] and population movement [32]. In [18], this impact was measured by determining the competition effectiveness of the different parasite strains [drug sensitive (RS), partially resistant (R1) and fully resistant (R2)] when exposed to different drug situations. However, the model in [18] did not consider movement between populations with different transmission rates.

To compute parasite fitness, the key assumption is that fitness is directly proportional to: (i) the duration of infection from symptoms until clearance by drugs which takes 1*∕a* days, or by the host’s immune system, which takes 1*∕g* days; (ii) the number of human hosts available for infection; (iii) the transmission efficiency of each population. The latter is assumed to be the same for the three parasite strains (RS, R1, R2). This method of assessing parasite fitness will yield an equivalent formulation to that in [3], where fitness of a genotype over a malaria generation was defined as a product of (i) its expected lifespan before it is cleared by drugs, or the host’s immune system, (ii) the number of potentially successful secondary transmissions, and (iii) the proportion of these secondary inoculations that are introduced into individuals who can be infected by the parasite of the given genotype (strain).

In a human population, the duration of a malaria parasite infection is the mean lifetime of the infection in non-immune humans (denoted ) and in semi-immune humans (denoted  ), each appropriately weighted by the proportion of infections that occur in each group. Recall that the index *i* refers to whether the population is a high (*i* = *H*) or low (*i* = *L*) transmission area. If  is the proportion of infections that occur in semi-immune individuals, then  is the proportion in non-immune individuals. Hence, the mean life-time of an infection in a human population in the high , and low  transmission regions are
2H2L

respectively. In equations (2H) and (2L),  and  are the mean lifetime of a malaria parasite infection in non- and semi-immune individuals, defined as
34

The term  represents the proportion (*λρ*) of non-immune infected treated individuals whose parasites are immediately cleared via treatment after 1*∕a* days, while  represents the proportion (*λ*^'^*ρ*) in semi-immune individuals. The remaining fractions of untreated infections, (*1* - *λρ*) for non-immune humans, and (*1* - *λ* ' *ρ*) for semi-immune humans, have their infection either cleared by the immune system or via treatment. For non-immune humans, the fraction  of untreated infections are cleared by the immune system at the average clearance rate of , while the remaining fraction , are cleared via treatment after entering the treated class some 1*∕a* days later. In semi-immune individuals, the fraction of untreated infections that are cleared by the immune system at the average clearance rate of  while the fraction that are cleared via treatment some 1*∕a* days later after entering the treated class is .

To compute the fitness of each parasite strain [drug-sensitive (RS), partially resistant (R1) and fully resistant (R2)], the populations that each can infect need to be known. All three strains (RS, R1, R2) can infect susceptible humans with no residual drug levels in their blood stream (i.e. *S*, *S*_*a*_), as well as a proportion *q* of infected but untreated humans (*qA, qA*_*a*_) with no drugs in their blood stream. Partially resistant (R1) and fully resistant (R2) parasites can additionally infect those treated humans with residual drugs in their blood stream but with no stimulated immune responses i.e. the individuals in classes *T*_2_^′^ and (1 - *b*)*T*_3_^′^, but not the *T*_1_ or *T*_1_^′^ individuals because of their development of temporary immunity. Fully resistant parasites (R2) can additionally infect any treated humans regardless of their drug titer. Therefore, they can additionally infect the *T*_2_ and *T*_3_ individuals. Thus, the combined proportion of humans infected by RS, R1 and R2 parasites are respectively, . Since a fully resistant treatment can only be cleared by the immune system, its lifetime is 1*∕g*. Therefore, the fitness of each parasite strain, respectively, becomes
5

The term *k* is the number of potentially successful secondary transmissions assumed to be the same for each parasite strain (since transmission efficiency is assumed to be identical). Assuming that partially resistant parasites replace susceptible types and fully resistant types replace partially resistant types, the relative fitness (*F*), will be the ratio of the more resistant parasite type over the more sensitive type and can be computed as
67

where  is as defined in equation (2H) when in the high transmission area, and as in equation (2L) when in the low transmission area. The percent of spread per parasite generation is then
8

In computing the fitnesses of each parasite population, equations (IH) and (1L) are solved to obtain the equilibrium values of the state variables, as described in Table 1. These equilibrium values, representing disease status for the human dosing model, are then used in equation (5) to determine the fitnesses of each parasite strain. The model equations are solved numerically in R (version 3.0.1) [33] using the lsoda numerical solver from the odesolve package. The lsoda function calls the Fortran function of the same name from the ODEPACK and uses Adams method and Backward Differentiation Formula for solving nonstiff and stiff equations [34, 35].

**Table 1:** **State variables and their description**

State variables	Description of state variables
*S*	Susceptible Untreated Non-immunes
*S*_*a*_	Susceptible Untreated Semi-immunes
*D*	Symptomatic Infected and Treated Non-immunes
*D*_*a*_	Symptomatic Infected and Treated Semi-immunes
*A*	Infected Untreated Non-immunes
*A*_*a*_	Infected Untreated Semi-immunes
*P*	Uninfected Non-immunes with Temporary Immunity
*P*_*a*_	Uninfected Semi-immunes with Temporary Immunity
*T*_1_ *, T*_1_^′^	Symptomatic Infected and Treated Non-immunes with Drug in bloodstream
*T*_*a*_ *, T*_*a*_^’^	Symptomatic Infected and Treated Semi-immunes with Drug in bloodstream
*T*_2_ *, T*_2_^′^	Susceptible IPT treated Non-immunes with Drug in bloodstream
*T*_3_ *, T*_3_^′^	Infected and IPT Treated Non-immunes with Drug in bloodstream

## Results

### Movement *versus*no movement: effect of IPT on the rate of spread of resistance

**Figure 2:**
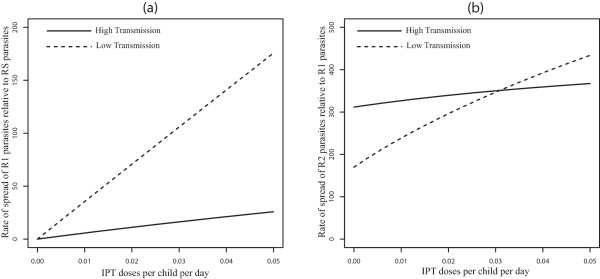
**No movement - effect of IPT coverage.** Model results without movement, *p*_12_ = *p*_21_ = 0, showing the effect of increasing IPT coverage with SP on the percent increase of R1 relative to RS and R2 relative to R1 resistance. Graph **(a)** represents the rate of spread (in percent) of R1 relative to RS parasites, while graph **(b)** represents that of R2 relative to R1. The parameter *p*_12_ is the rate of movement from the high malaria transmission area to the low transmission area, and *p*_21_ is the rate from the low transmission area to the high transmission area.

When movement rates are set to zero (*p*_*12*_ = *p*_*21*_ = *0*), the results produced in [18] are recovered (see Figure 2a and b). The rate of spread of R1 (partially resistant) parasites relative to RS (susceptible) parasites increases as IPT dose frequency increase, and this increase is more significant in the low transmission region (Figure 2a). This is in alignment with evolutionary expectations. R1 parasites are expected to spread faster than RS parasites because R1 parasites can infect a broader class of hosts. This equates with higher relative fitness fitness [eqn. (7)] and a faster rate of spread [eqn. (8)]. Faster rate of spread of the R1 parasites in the low-transmission population results from there being fewer treated individuals and more individuals in the susceptible and untreated classes, *S* and *A*, when transmission is low (see Table 2). As a consequence, more individuals in these *S* and *A* classes receive IPTi which can protect them against the malaria parasites. Eventually the level of anti-malarial drug in these individuals declines to sub-therapeutic levels such that it is no longer effective against the R1-type parasites, but still suppresses the growth of RS parasites. This is included in the model as the transmission from the IPTi treated classes *T*_2_ and *T*_3_, to the respective classes  and , that represent those with reduced levels (sub-therapeutic levels) of anti-malarial drugs in their systems (See Figure 1). Since the individuals in  and  classes can be infected by R1 parasites, the R1 parasites experience higher fitness when compared to RS parasites which cannot infect individuals from  and  classes. As IPT dose frequency increases, selection against RS parasites also increases, resulting in a net relative advantage for partially resistant parasites.

**Table 2:** **Equilibrium proportions of individuals in each state with and without movement between areas of low and high transmission**

Description	State	Without movement		With movement	
		Low	High	Low	High
Uninfected and untreated	*S*	34.269%	0.027%	1.265%	0.079%
	*S*_*a*_	2.725%	0.234%	7.414%	0.363%
Infected but untreated	*A*	3.156%	0.111%	0.161%	0.307%
	*A*_*a*_	0.310%	2.658%	1.439%	3.533%
Immune-protected but uninfected	*P*	7.949%	0.539%	0.973%	1.042%
	*P*_*a*_	10.137%	86.450%	79.764%	82.184%
Infected and treated	*D*	0.824%	0.103%	0.054%	0.270%
	*D*_*a*_	0.089%	0.765%	0.420%	1.011%
Infected prior to treatment	*T*_1_	8.550%	1.063%	1.279%	2.087%
with drug in bloodstream	*T*_3_	1.310%	0.092%	0.172%	0.216%
Uninfected prior to treatment	*T*_*a*_	0.925%	7.936%	6.324%	8.528%
with drug in bloodstream	*T*_2_	28.450%	0.022%	0.735%	0.380%

All results are based on model parameters as summarized in Additional file 2: Table S3, with *p*_21_ */p*_21_ = 1 = *m*, *p*12 = 0*.*02. Note that the values for *T*_1_, *T*_2_, *T*_3_ and *T*_*a*_ represent the sum of *T*_1_ and *T'*_1_, *T*_2_ and *T'*_2_, *T*_3_ and *T'*_3_, and *T*_*a*_ and *T'*_*a*_ respectively.

Under the no-movement assumption, the results also indicate that R1 parasites spread faster in low transmission areas than in high transmission areas at all IPT dosage frequency (Figure 2a). In addition, once resistance has emerged, the rate of spread of fully resistant (R2) parasites relative to R1 parasites was not significantly affected in high transmission areas with increase in the frequency of IPT dose (Figure 2b). However, the rate in the low transmission area increased with increasing IPT dosage frequency, but is initially lower than the rate in the high transmission area, switching at c = 0.03 days^-1^ as IPT dose frequency increased. In the high transmission region, the majority of the individuals at equilibrium are immune protected (see Table 2) and do not receive IPT, captured by the smaller change effect in the rate of spread of R1 relative to RS and R2 relative to R1 parasites as IPT dosing rate increase (Figure 2a and b). In the low transmission area, as the rate of IPT dosing increase, the proportion of treated individuals  increase, increasing the fitness of the R2 parasites and the fitness landscape shift in favor of high parasite fitness in low transmission areas. The proportion of treated individuals also increase with increasing IPT dosage in high transmission areas. However, the classes of treated hosts are a relatively smaller fraction of the population and there isn’t as much room for expansion of R2-susceptible hosts in these classes. As a result, the portion of R2 fitness increase attributable to these classes does not increase as quickly and is eventually surpassed by the more rapid gains accrued from the increase in the  classes in the low transmission area.

**Figure 3:**
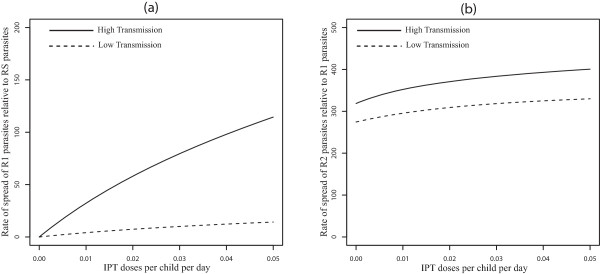
**Symmetric movement - effect of IPT coverage for 50 days stay.** Model results with symmetric movement and equal population sizes (*m* =1, *p*_12_ */p*_21_ = 1, *p*_21_ = 0*.*02 *per day*), showing the effect of increasing IPT coverage with SP on the percent increase of R1 relative to RS (Graph **(a)**) and R2 relative to R1 (Graph **(b)**) resistance. The parameter *p*_12_ is the rate of movement from the high transmission area to the low transmission area, and *p*_21_ is the rate from the low transmission area to the high transmission area. , is the ratio of the high transmission population to that of low transmission population.

The effects of movement between low and high transmission areas is to switch which malaria resistant-strain spreads fastest relative to the no-movement scenario. With symmetric movement between equal sized populations , the rate of spread of R1 relative to RS parasites is faster in the high transmission area (Figure 3a). This is a reversal of the results produced without movement (see Figure 2a). Without movement, the IPT treated classes  represent a larger portion of the low-transmission population relative to the high transmission population (Table 2). Since these classes can be infected by R1 but not RS, R1 is expected to spread faster in the low transmission area. However, when movement is added the portion of treatment classes is larger in the high transmission areas relative to low transmission areas (Table 2). This makes the high transmission areas more suitable for the spread of R1 parasites relative to RS parasites.

**Figure 4:**
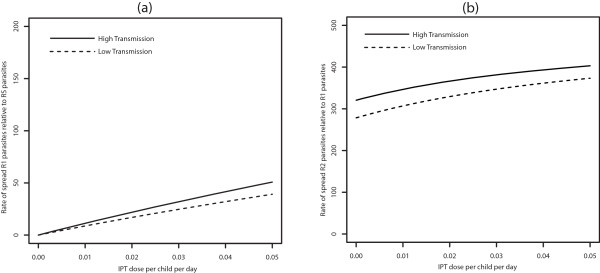
**Symmetric movement - effect of IPT coverage for 1 day stay.** Model results with symmetric movement and equal population sizes for 1 day visitation (*p*_21_ = 1*, p*_21_ */p*_12_ = 1 = *m*) showing the effect of increasing IPT coverage with SP on the percent increase of R1 relative to RS (Graph **(a)**) and R2 relative to R1 resistance (Graph **(b)**).

Furthermore, with movement, the switch from faster spread of R2 relative to R1 in high transmission areas to faster spread in low transmission areas (Figure 2b) disappears, and the relative rate of spread of R2 to R1 is always higher in the high transmission area (See Figure 3b). The qualitative results are the same across a range of movement rate ratios for equal sized populations (*m* = *p*_21_ = *p*_12_ = 1) in the low and high transmission areas [See Figure 4a and b for *p*_*21*_ = *1* per day, and Figure 1a and b (See Additional file 2) for *p*_*21*_ = 0.1 *per day*]. This suggests that the switch in the order of where R2 spreads faster (high *vs* low transmission) is robust to changes in the assumptions about the rates of movement. The other point to note in these figures is that, without movement the switch from faster R2 spread in the high transmission area to faster R2 spread in the low transmission area is almost completely the result of a rapid increase in the rate of R2 spread in low transmission areas as the rate of IPT dosing is increased. However, when the effects of movement are added the variation in the rate of R2 spread with dosing rate is largely muted, resulting in the simpler relationship between low and high transmission areas and the rate of R2 spread.

The differences seen can be explained using Table 2, which shows the quantitative values of the proportion of individuals in each compartment at equilibrium. A presumptive dosing frequency of about one dose every 60 days (c = 0.016 per day) was assumed as in [18] while other parameters remained the same and are shown in Additional file 2: Table S3. The values for *T*_1_, *T*_2_, *T*_3_ and *T*_*a*_ (Table 2) represent the sum of *T*_1_ and *T'*_1_, *T*_2_ and *T'*_2_, *T*_3_ and *T'*_3_ and *T*_*a*_ and *T'*_*a*_ respectively. From the values and equation (8), with symmetric movement between equal sized populations (where *m* = *p*_*21*_/*p*_*12*_ = *1*, *p*_21_ = 0.02 per day), R1 parasites have the potential to increase by 46.3% each generation relative to RS parasites in the high transmission area, but only by 6.2% in the low transmission area. Without human movement, the increase is only 9.05% in the high transmission area, but 59.08% in low transmission area. The switch is influenced by the large decrease in the proportion of the population residing in the non-immune categories in low transmission area when movement is included.

### Movement *versus*no movement: effect of drug elimination time on the rate of spread of resistance

SP and CPG-DDS are two treatment lines used for uncomplicated *Plasmodium falciparum* malaria [3, 18, 36]. As earlier noted, SP contains pyrimethamine and sulphadoxine which both have longer half-lives [22, 37] than dapsone and chlorproguanil in CPG-DDS [23, 36]. Here, the effect of drug elimination time on the spread of resistance when movement is considered is investigated.

**Figure 5:**
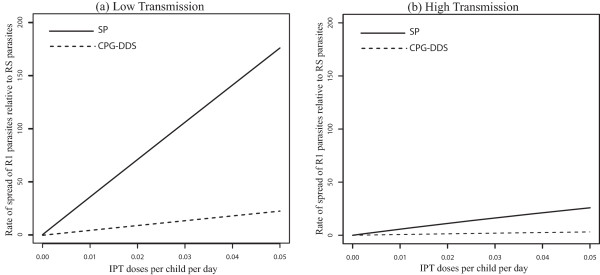
**No movement - effect of drug half-life.** Effect of IPT treatments with drugs with different half-lives, SP or CPG-DDS, on the increase percentage of R1 relative to RS in both a low (Graph **(a)**) and high (Graph **(b)**) transmission setting when there is no movement (*p*_12_ = *p*_21_ = 0). Compare Graph **(a)** with Figure 6 of O’Meara et al. [18].

**Figure 6:**
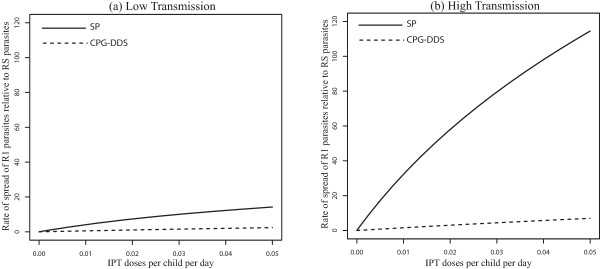
**Symmetric movement - effect of drug half-life for 100 days stay.** Effect of IPT treatments with drugs with different half-lives, SP or CPG-DDS, on the increase percentage of R1 relative to RS in both a low (Graph **(a)**) and high (Graph **(b)**) transmission setting, when symmetric movement between equally sized populations (m = *p*_12_ */ p*_21_ = 1*, p*_12_ = 0*.*01) is considered. The parameter *p*_12_ is the rate of movement from the high transmission area to the low transmission area, and *p*_21_ is the rate from the low transmission area to the high transmission area, , is the ratio of the high transmission population to that of low transmission population.

When the use of the drugs SP and CPG-DDS for IPT and for clinical treatment are compared, it is observed that the inclusion of symmetric movement between low and high transmission areas did not qualitatively change the potential spread of drug resistance by drugs with longer half-lives, in both the low and high transmission areas (see Figures 5 and 6). Regardless of movement, drugs with longer half-lives have a greater potential of promoting the evolution of drug resistance (R1 relative to RS, and R2 relative to R1) than those with shorter half-lives. This makes sense biologically since the persistence of drugs at sub-therapeutic concentrations will provide a wider opportunity for parasites to “learn to live with the drug” i.e. a wider window for selection of drug-resistant mutations. Thus the proportion of treated individuals  will be higher in the populations, giving a higher fitness advantage to R1 parasites relative to RS parasites. Since R2 parasites can additionally infect *T*_*i2*_ and *T*_*i3*_ individuals, they have a slight fitness advantage over R1 parasites. However, because in high transmission regions very few treatments occur, this effect is not large. Moreover, note that regardless of movement, CPG-DDS, the drug with shorter half-life had a lower potential for the spread of partial resistance in both the low and high transmission areas.

Furthermore, movement decreases the rate of spread of resistance of R1 relative to RS parasites significantly for both drugs in the low-transmission area (compare Figures 5a and 6a) while it slightly increases the rate of spread for the drug with longer half-life in the high-transmission area (compare Figures 5b and 6b). With symmetric movement, between the two populations such that *p*_*12*_/*p*_*21*_ = 1,  *p*_*12*_ = *0.01* per day, the disease demography of the low transmission region reflect that of the high transmission region, with more immune-protected individuals in both transmission regions, and thus not receiving IPT. Hence the pool of treated individuals (*T*_*i*2_, *T'*_*i*2_, *T'*_3_ and *T'*_3_) is generally smaller, but is slightly higher in the high transmission region than in the low transmission region, giving a higher fitness advantage to R2 parasites over the R1 parasites. Notably, the slower rate of spread in the low transmission region is significant from a control perspective because both drugs can be useful for a much longer period of time.

**Figure 7:**
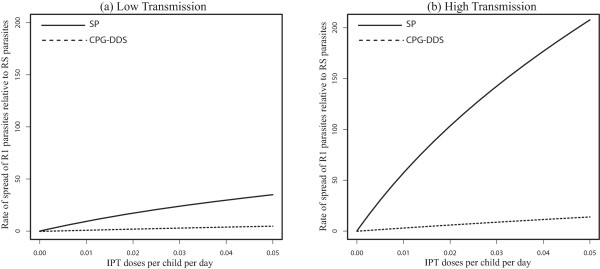
**Non symmetric movement with m < 1 - effect of drug half-life for 100 days stay.** Effect of IPT treatments with drugs with different half-lives, SP or CPG-DDS, on the increase percentage of R1 relative to RS in both a low (Graph **(a)**) and high (Graph **(b)**) transmission setting when *p*_21_ */p*_12_ = 0.5 = *m, p*_12_ = 0*.*01.

**Figure 8:**
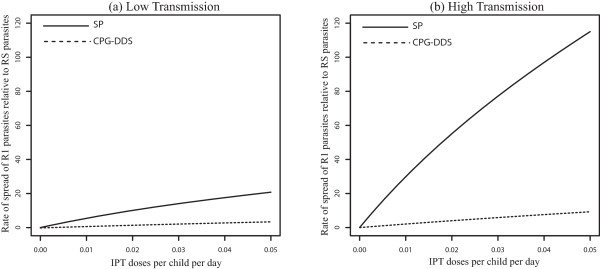
**Non symmetric movement with m > 1 - effect of drug half-life for 100 days stay.** Effect of IPT treatments with drugs with different half-lives, SP or CPG-DDS, on the increase percentage of R1 relative to RS in both a low (Graph **(a)**) and high (Graph **(b)**) transmission setting when *p*_21_ */p*_12_ = 1.5 = *m , p*_12_ = 0*.*01.

When the results are compared for different sizes of the total population, *m* ≠ *1*, the rate of spread in the high transmission area is affected by the size of the low transmission population. For example, if the low transmission area has a population size that is twice that of the high transmission area (*m* = *0.5*), the rate of spread in the high transmission area is much higher, leading to a slightly higher rate of spread of R1 relative to RS parasites in the low transmission area (compare Figures 6 and 7). This is true for any *m* < *1*. However, this result will depend on the movement rates used. For, *m* > *1*, the high transmission population size is larger and the interplay between the high and low transmission areas results in a lower potential rate of spread of R1 parasites in the low transmission area (see Figure 8), even more so than in the high transmission area, depending on the movement rates.

### Movement *versu*s no movement: effect of treatment on the rate of spread of resistance

**Figure 9:**
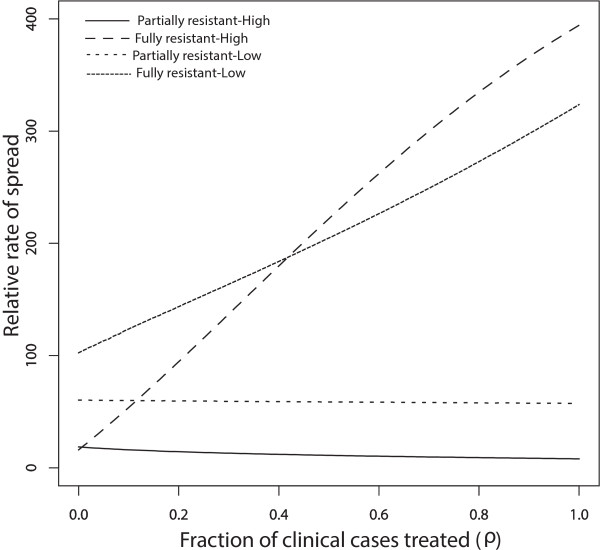
**No movement-effect of treatment proportions.** The effect of treatment on the rate of spread (in percent) of resistance when there is no movement (*p*_12_ = *p*_21_ = 0). Compare with Figure 7 in [18].

Drug treatment of infected and uninfected non-immune humans plays a crucial role in the spread of partially resistant (R1) parasites [3, 18, 38] (see Figure 2 for the case when there is no movement). On the other hand, the long duration of infections that are resistant to drug treatment and the availability of larger proportions of humans to fully resistant (R2) parasites are the driving forces behind the spread of fully resistant (R2) parasites [18]. Treating more infectious humans (larger *ρ*) reduces the average duration of drug-sensitive and partially resistant infections. However, this has no effect on fully resistant infections because they are refractory to treatment. Therefore, as *ρ* increases, the average lifetime of R1 parasites reduce while that of R2 is not affected, leading to an increase in the relative fitness of R2 to R1 parasites, meanwhile the relative fitness of R1 to RS parasites remains unchanged [this follows from equations (6) and (7) and Figure 9]. Note that without movement, for small proportions of *ρ*, the potential spread of R2 parasites in low transmission areas is initially larger than in high transmission area, but switches as *ρ* increases. However, the spread of R1 relative to RS is faster in low transmission area than the relative spread in high transmission areas for all values of *ρ*.

**Figure 10:**
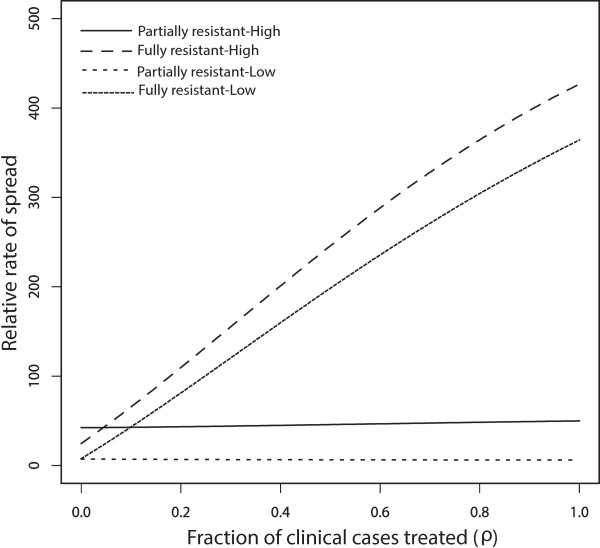
**Symmetric movement-effect of treatment proportions for 100 days stay.** Effect of treatment on the rate of spread (in percent) of resistance when considering symmetric movement between the high and low transmission areas so that *m* =1 *= p*_21_ */p*_12_ *, p*_12_ = 0*.*01.

When symmetric movement is included with *m* =1 and *p*_12_ = *p*_21_ = 0*.*01, the spread of R2 relative to R1 parasites increases as *ρ* increases both in the high and low transmission areas, but the spread is faster in high transmission area for all values of *ρ* (Figure 10). This result is corroborated by Figure 3 which shows that with symmetric movement between the high and low transmission areas, the rates of spread of R1 relative to RS parasites and R2 relative to R1 parasites are both higher in the high transmission areas.

**Figure 11:**
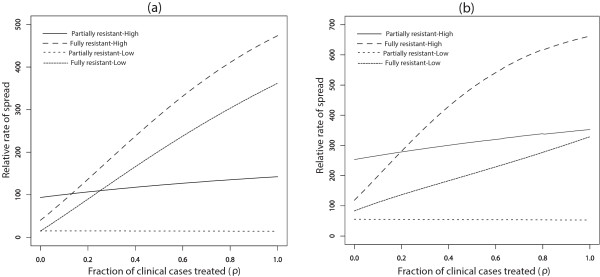
**Non symmetric movement-effect of treatment proportions for 100 days stay.** Effect of treatment on the rate of spread (in percent) of resistance. Graph **(a)** corresponds to the case when*, m =0.5 = p*_21_ */p*_12_ *, p*_12_ = 0*.*01, while Graph **(b)** corresponds to the case when *m* =0*.*1 = *p*_21_ */p*_12_ *, p*_12_ = 0*.*01.

The effect of having a larger low transmission population than the linked high transmission population (*m <*1) is exhibited through increasingly higher relative rates of spread of partial and full resistances in the high transmission area. For example, for two values of *m <*1 (*m* = *p*_21_/*p*_12_ = *0.5* with *p*_12_ = 0*.*01 per day, and *m* = *p*_21_/*p*_12_ = *0.1* with *p*_12_ = 0*.*01 per day), the rate of spread of R2 relative to R1 and R1 relative to RS are each larger in the high transmission area than the corresponding relative rate of spread in the low transmission area (see Figure 11a for *m* = 0*.*5 and Figure 11b for *m* =0*.*1).

### Implications of slow and fast movement rates on the rate of spread of resistance

In the model results presented so far, movement rates of *p*_*ij*_ = *0.01* and *p*_*ij*_ = *0.02* per day (i.e. 100 days and 50 days visitation respectively) were utilized. A discussion is now presented on how shorter length of visitation may impact the results outcome, and regions were short and long movement rates might be applicable. Recall that the present results are based on the assumption that the populations are in dispersal equilibrium.

The East African Highlands are an example of a region that depicts longer and shorter term movement patterns [39]. Highlands in malaria endemic regions tend to have low and unstable malaria transmission intensities [8–10], compared to the high transmission, endemic lowlands. The highlands are fertile with lots of agricultural activity, and are usually the most densely populated areas in the region, with significant movement between the highlands and the lowlands. People move between these areas to sell their agricultural products/trade, visit relatives, go to school, etc. Besides the highlands, urban areas typically have lower malaria transmission intensities than rural areas. This is mostly because compared to rural areas, urban areas usually have better housing and drainage systems, fewer swamps, and better access to health care interventions. Frequent population movement between rural and urban areas also provides connectivity between the two different malaria transmission settings. For example, Nairobi, the capital city of Kenya, is a low malaria transmission area with significant daily movement between the city and the high transmission Lake Victoria region [40]. Commuters, tourists, short-term workers, and other individuals moving from rural to neighboring urban areas may carry parasites and mosquitoes which may breed in pools. The time period spent in these regions may vary from days to months at a time [39]. Thus a movement period with visitors spending an average of up to 100 days is not unusual. When the period is in the order of days, the two regions have stronger interaction—this is common in regions where the movement is more for agricultural purposes. Periods that are in the order of months will reflect regions were movement is more for short-term work and schooling purposes (for example boarding schools).

**Figure 12:**
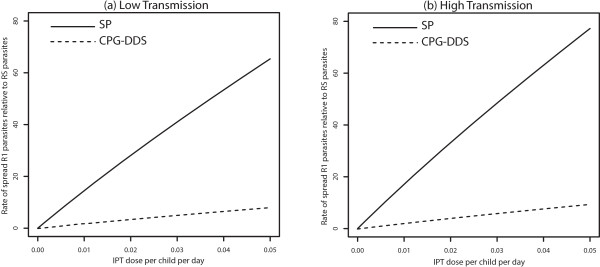
**Symmetric movement - effect of drug half-life for 1 day stay.** Effect of IPT treatment with different half-live drugs, SP or CPG-DDS, on the increase percentage of R1 relative to RS in both a low (Graph **(a)**) and high (Graph **(b)**) transmission setting when *p*_21_ */p*_12_ = 1 = *m, p*_12_ = 1 (i.e. a one day visitation).

**Figure 13:**
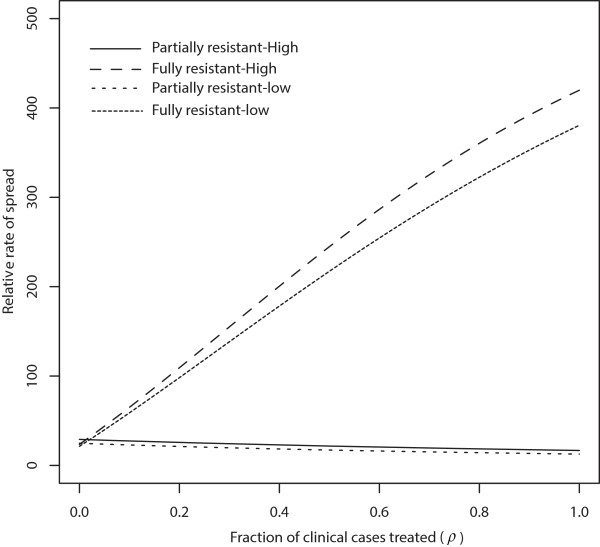
**Symmetric movement - effect of treatment proportions for 1 day stay.** Effect of treatment on the rate of spread (in percent) of resistance when we consider symmetric movement between the high and low transmission areas so that *m* =1 *= p*_21_ */p*_12_ = 1*, p*_12_ = 1.

When the low and high transmission regions are strongly connected with fast movement rates between the two, there is little distinction between the two regions in relation to the relative rate of spread of R1 to RS parasites and R2 to R1 parasites. For example, for a one day visitation with symmetric movement between the low and high transmission regions (*p*_12_/*p*_21_ = 1 with *p*_12_ = 1 per day), the effect of increasing IPT coverage with SP on the percent increase of R1 relative to RS parasites and R2 relative to R1 parasites is similar in the high and low transmission area (Figure 4), and reflected the behaviour of the high transmission area. This can be expected since the turn over between the populations is high. The distinction becomes increasingly evident as the turnover in each population is reduced due to a slower movement rate, as indicated on Figure 3. Similarly, for a shorter visitation period, the effect of IPT treatments with drugs with different half-lives and also the effect of treatment on the spread of resistance were similar in low and high transmission regions (see for example Figures 12 and 13, respectively).

Overall, the fast movement rates lead to fast turnover within each population. The net effect is that the interaction between the populations is strong and hence the rate of spread of R1 relative to RS and R2 relative to R1 parasites behave the same in both populations. It is worth noting that the qualitative results with human movement are different from the results without movement. Moreover, the results are applicable to interacting high and low transmission regions with fast or slow movement rates. Additionally, from the results, there is little qualitative distinction in the movement results between a 10 days and a 50 days or 100 days visitation. For graphs based on a 10 days visitation, see Additional file 2: Figures S1a, b, S2a, b and S3.

## Discussion

Given the increasing popularity of IPT, it is important to understand the role this malaria preventive strategy has on the spread of anti-malarial drug resistance, and provide pertinent information that may help guide national public health policies. Because public health policies impact the health status of individuals within communities, they may not necessarily always be a one-size-fit-all, hence need to be updated or changed with respect to local dynamics. Thus, policies can only remain in place as long they are acceptable, sustainable and positively impacting the health status of the community. For example, a number of countries in Africa and elsewhere have over the years had to change their malaria treatment policies when the drugs became largely useless due to drug resistance [41].

There are many factors that influence public health formulation and change, including but not limited to: disease burden and mortality rates, treatment and treatment efficacy factors, economic factors, political factors, community beliefs factors, factors relating to changes in community landscape and dynamics, and legislative issues. Research data and mathematical models are important because they provide a compelling framework for most policy models, which may include changes to drug policies and treatment regimens, as well as disease and drug-resistance control measures [41–44].

In the present mathematical model, the inclusion of movement between neighbouring low and high transmission areas in malaria endemic regions suggests different control policies when monitoring the rate of spread of drug resistance. The measure of how much faster resistance spreads, and whether spread is expected to be faster in high or low transmission areas are thus important features to investigate.

The model results consistently indicate a faster rate of spread for malaria parasites with higher degrees of resistance. This is because in the present model, resistance to treatment is associated with higher fitness and fitness is associated with the ability to infect humans in a larger number of classes. Drug-susceptible parasites can only infect hosts that are not receiving treatment and who do not have any level of acquired immune response. Partially resistant parasites can infect all hosts subject to infection by the non-resistant parasites as well as hosts that have sub-therapeutic anti-malarial drug concentrations following treatment. The range of hosts that fully resistant parasites can infect is even wider and includes those with full therapeutic doses as well as those with some partial immunity.

The fitness measure used in the present model to assess the rate of parasite and hence disease spread, is associated with between host transmission. There is also a fitness component associated with potential competition between parasite strains within a single host. Often drug resistant genes are associated with a reduction in parasite fitness in the absence of the corresponding anti-malarial drug. However, these fitness costs are often offset by compensatory mutations. As a result, fitness costs associated with resistant genes is often short lived (this assumption may not be true when there is compliance to the anti-malarial drug in the population, and in situations where there is prolonged and frequent use of anti-malarial drugs). Since the present model is focused on the long-term equilibrium dynamics of disease spread, the effects of temporary fitness costs were not incorporated.

Model results indicate that movement between low and high transmission areas may invert the expected role of low and high transmission areas in the spread of anti-malarial drug resistance. When movement occurs between high and low transmission areas, resistance spreads more quickly in high transmission areas and does so at all rates of IPTi dosing and in both the RS *vs* R1 and R1 *vs* R2 competition (Figure 3a and b). The results are robust to changes in both the strength of connectivity between the two transmission areas, as well as to changes in the relative size of the two areas.

However, there is a minimal movement rate required to qualitative change the rates of spread in the non-spatial result in [18]. In the case of symmetric movement, a movement rate with *p*_12_ = *p*_21_ = 0.00015 *per day* (about an 18-year period) was necessary to see a qualitative change in the non-spatial/no movement result of [18], in both the low and high transmission regions. More specifically, with symmetric movement, *p*_12_ (=*p*_21_) has to be at least 0.0001 per day (i.e. in the order of about 27 years) in order to lose the switch observed in the percent rate of spread of R2 relative to R1 produced by the non-spatial model (Figure 2a and b). For *p*_12_ = *p*_21_ = 0.0001 per day, the switch produced in the non-spatial model (of Figure 2b), which occurred at roughly c =0.03 per day, is lost with movement at c slightly above 0.05 per day (See Additional file 2: Figure S4). Thus, with symmetric movement and *p*_12_ = *p*_21_ = 0.0001 per day, the rate of spread of R2 relative to R1 parasites is always higher in the high transmission region for IPT dosing frequency lower than c =0.05 per day (i.e. once every 20 days). However, the rate of spread of R1 relative to RS is still higher in the low transmission region, than in the high transmission region, as in the non-spatial model, but the difference is not very significant. Increasing the movement rate to a rate slightly higher than 0.00015 per day (i.e. about an 18-year period) but lower than 0.0002 per day (i.e. about a 14-year period) initiated the change in the low and high transmission region, leading to a higher percent rate of spread of R1 relative to RS in the high transmission region. Hence, the movement rate that is required to produce a qualitative change in the non-spatial results in both the low and high transmission regions is slightly greater 0.0015 (see Additional file 2: Figures S5 and S6), when symmetric movement is considered.

In the presence of IPTi, the rapid spread of highly resistant parasites in high transmission areas could be due to at least two factors: (i) chemotherapy eliminates the competing sensitive parasites, giving a great selective advantage to the resistant parasites, with resultant high levels of gametocytes; the infective stage for mosquitoes; (ii) the large number of mosquitoes in high transmission areas compared to the low transmission areas; ensuring faster spread of the parasites (see [2]).

These results have potentially important public health applications for the monitoring policies looking for the emergence and spread of resistant parasites. If regions are connected with each other, the present results suggest that monitoring in high transmission areas is likely to detect the fastest spread of drug resistance regardless of the rate of IPTi dose applied and independent of which level of resistance is being detected. On the other hand, if regions are assumed to be, or in fact are, unconnected, then the best place to monitor in order to detect the rapid spread of resistance depends on both the rate of IPT dose (Figure 2b) and the level of resistance that is to be detected (Figure 2a and b). It remains to be seen how these results corroborate with field studies.

Model results also indicate that the demography of infection in low transmission areas tends to change to reflect the demography of high transmission areas, when regions are connected by movement (Table 2). This differs from the result in [18]. When regions are isolated, most individuals in low transmission areas (>80%) are uninfected, with larger but closely similar percentages either under prophylactic treatment (*T*_2_, 28.45%) or under the untreated susceptible group (*S*, 34.27%), and smaller percentages under classes *P* (7.95%) and *P*_*a*_ (10.14%). In high transmission areas, the large majority of the population is in class *P*_*a*_ (86.45%). When regions are connected, low transmission areas experience a large demographic shift and come to reflect the composition of the high transmission areas with the majority of the population in class *P*_*a*_ (79.76%), while the demography of the high transmission area changes very little.

In this model, analysis on treatment involved the drugs SP and CPG-DDS. Malaria parasites in various areas are exhibiting resistance to SP [12], and thus it is no longer a front-line drug. Furthermore, recent articles [45] cited issues relating to CPG-DDS. However, the present model can be applied to other anti-malarial drugs with long, intermediate or short half-lives. Additional file 2: Figure S7 shows the qualitative results for SP when compared with a drug whose half-life is four times that of CPG-DDS (but lower than that of SP). The curve for this drug produced graphical results with a curve between that of the CPG-DDS and SP curves. In general, if a drug has a half-life close to that of SP, with all other factors held constant, the expectation is that the curves for this drug would be close to that of the SP curves, and if the half-life is closer to that of the CPG-DDS drug, the results would be closer to the CPG-DDS graphs.

Overall, results from this model suggest that when monitoring the spread of drug resistance, it is important to consider both the geographical landscape and social aspects linking two transmission areas. The results can be extended to consider pulse timing methods in the administration of IPTi, where IPTi is administered at specific regularly pulsed intervals of either three months, six months or some appropriate time period. This may require formulating a model that explicitly accounts for the different human disease classes, and is currently under investigation.

The modelling paradigm used here is different from most modelling paradigms of malaria. In studying malaria, focus can be placed on aspects involving one, two or all three of the following interacting components related to malaria transmission and success: the human, that hosts the parasite; the mosquito vector which hosts part of the parasite’s life-cycle and responsible for transmitting the parasite from one human to another; and the parasite, the agent that causes the disease [46]. Models for malaria have mostly focused on the transmission dynamics of the disease, where the relationship between the humans and the mosquitoes, in which the parasite determines the status of each human or vector classes, has been carried out (e.g. [47–49]). Such models usually require stability methods and analyses. Other models have emphasized the life-cycle of the mosquito (for example [50–54]), while others have placed focus on the parasite component (e.g. [55, 56]). The present model focuses on the parasites and uses fitness functions, determined by how many humans the parasites can infect, to investigate how fast resistance may spread within neighbouring communities when the populations are at endemic equilibrium. Thus, parasites were tracked in a scenario where the human populations are already at equilibrium.

## Conclusions and recommendations

In conclusion, when human movement frequently occurs between areas with different malaria transmission intensities:

-  the expected role of low and high transmission areas in the spread of anti-malarial parasite resistance may be inverted, with resistance consistently spreading more quickly in high transmission areas for all rates of IPTi dosing and in both the RS *vs* R1 and R1 *vs* R2 competition;
-  the demography of infection in low transmission areas tends to change to reflect the demography of high transmission areas when regions are connected by movement;
-  fast movement rates lead to strong interaction between the interacting populations and the rate of spread of R1 relative to RS and R2 relative to R1 parasites tend to behave the same in both populations, as should be expected.

Results from this model have important public health applications for the monitoring/surveillance policies looking for the speed of spread of resistant parasites. If regions are connected with each other by human movement, monitoring in high malaria transmission areas is likely to detect the fastest spread of drug resistance, regardless of the rate of IPTi dose applied and independent of which level of resistance is being detected. On the other hand, if regions are assumed to be, or in fact are, unconnected, then the best place to monitor in order to detect the rapid spread of resistance depends on both the rate/frequency of IPT dose and the level of resistance that is to be detected. Overall, these results indicate that different public health policies are needed when the area in question is an isolated high or low malaria transmission area, or whether it is close and interacting with a neighbouring low or high transmission area via human movement.

Furthermore, the same drug is sometimes utilized for IPTi and for symptomatic drug treatment of malaria. Thus it may be difficult to use experiments to distinguish the individual roles of either IPTi or symptomatic drug treatment on the acceleration of the spread of drug resistant malaria, with or without human movement. A theoretical framework, as presented here, is thus relevant as the effect of IPTi in accelerating the spread of drug resistance can be isolated as demonstrated in this study.

## Electronic supplementary material

Additional file 1: **This supplemental document contains the main model equations.** (PDF 48 KB)

Additional file 2: **This supplemental document contains supplemental figures showing results from the present spatial model for an average of a 10 days visitation when symmetric movement is considered.** The results show that, overall, the qualitative results of the rate of spread of resistance with increases in IPT dosage is the same across a range of movement rate ratios in the low and high transmission areas. There is little distinction between an average of a 10 days visitation, a 50 days visitation, and a 100 days visitation (compare [Additional file 2: Figures S1, S2 and S3] to Figures 3, 6 and 10). The same is true whether considering the effects of IPT treatment with drugs with different half-lives or the effect of treatment on the rate of spread of resistance. Additional file 2 also contains graphical results that indicate the movement rates required to effect change in the non-spatial model in [18] (see Figures 5, 6 and 7) as well results indicating the profile of the SP drug compared to a drug with half-life that is four times that of the CPG-DDS drug (Figure 8). Moreover, Additional file 2: Table S3, which gives the parameters, their description, and values used in the model analysis. (DOCX 932 KB)
