# Supplementary material for: The effect of intermittent preventive treatment on anti-malarial drug resistance spread in areas with population movement
Source: Malar J. 2014 Nov 15;13:428. doi: 10.1186/1475-2875-13-428 (PMC4289180; doi:10.1186/1475-2875-13-428)
Supplement: Supplementary file 1 — Additional file 1: This supplemental document contains the main model equations. (PDF 48 KB) [file 12936_2014_3653_MOESM1_ESM.pdf]

S1: Supplemental Document

Model Equations

Human Dosing Model with Movement in the High Transmission Area

$$
\begin{aligned}
\frac{dS_H}{dt} &= \mu - \Lambda_H S_H + \omega P_H + \sigma A_H - c S_H + (1-b) r_2 T'_{H3} + r_2 T_{H2} - \mu S_H + \frac{p_{21}}{m}[S_L - S_H] \\
\frac{dD_H}{dt} &= \lambda \rho \Lambda_H S_H - a D_H + (\nu + d\Lambda_H)\rho A_H - \mu D_H + \frac{p_{21}}{m}[D_L - D_H] \\
\frac{dA_H}{dt} &= (1-\lambda\rho)\Lambda_H S_H - g A_H - \sigma A_H - c A_H - (\nu + d\Lambda_H)\rho A_H - \mu A_H + \frac{p_{21}}{m}[A_L - A_H] \\
\frac{dT_{H1}}{dt} &= a D_H - r_1 T_{H1} - \mu T_{H1} + \frac{p_{21}}{m}[T_{L1} - T_{H1}] \\
\frac{dT'_{H1}}{dt} &= r_1 T_{H1} - r_2 T'_{H1} - \mu T'_{H1} + \frac{p_{21}}{m}[T'_{L1} - T'_{H1}] \\
\frac{dT_{H2}}{dt} &= c S_H - r_1 T_{H2} - \mu T_{H2} + \frac{p_{21}}{m}[T_{L2} - T_{H2}] \\
\frac{dT'_{H2}}{dt} &= r_1 T_{H2} - r_2 T'_{H2} - \mu T'_{H2} + \frac{p_{21}}{m}[T'_{L2} - T'_{H2}] \\
\frac{dT_{H3}}{dt} &= c A_H - r_1 T_{H3} - \mu T_{H3} + \frac{p_{21}}{m}[T_{L3} - T_{H3}], \qquad (1H) \\
\frac{dT'_{H3}}{dt} &= r_1 T_{H3} - r_2 T'_{H3} - \mu T'_{H3} + \frac{p_{21}}{m}[T'_{L3} - T'_{H3}] \\
\frac{dP_H}{dt} &= g A_H + r_2 T'_{H1} + b r_2 T'_{H3} - \omega P_H - \gamma \Lambda_H P_H - \mu P_H + \frac{p_{21}}{m}[P_L - P_H] \\
\frac{dS_{Ha}}{dt} &= \omega' P_{Ha} - \Lambda_H S_{Ha} - \mu S_{Ha} + \frac{p_{21}}{m}[S_{La} - S_{Ha}] \\
\frac{dD_{Ha}}{dt} &= \lambda' \rho \Lambda_H S_{Ha} - a D_{Ha} + \nu' \rho A_{Ha} - \mu D_{Ha} + \frac{p_{21}}{m}[D_{La} - D_{Ha}] \\
\frac{dA_{Ha}}{dt} &= (1-\lambda'\rho)\Lambda_H S_{Ha} - g A_{Ha} - \nu' \rho A_{Ha} - \mu A_{Ha} + \frac{p_{21}}{m}[A_{La} - A_{Ha}] \\
\frac{dT_{Ha}}{dt} &= a D_{Ha} - r_1 T_{Ha} - \mu T_{Ha} + \frac{p_{21}}{m}[T_{La} - T_{Ha}] \\
\frac{dT'_{Ha}}{dt} &= r_1 T_{Ha} - r_2 T'_{Ha} - \mu T'_{Ha} + \frac{p_{21}}{m}[T'_{La} - T'_{Ha}] \\
\frac{dP_{Ha}}{dt} &= \gamma \Lambda_H P_H + r_2 T'_{Ha} + g A_{Ha} - \omega' P_{Ha} - \mu P_{Ha} + \frac{p_{21}}{m}[P_{La} - P_{Ha}]
\end{aligned}
$$

Human Dosing Model with Movement in the Low Transmission Area

$$
\begin{aligned}
\frac{dS_L}{dt} &= \mu - \Lambda_L S_L + \omega P_L + \sigma A_L - cS_L + (1-b)r_2 T_{L3}^{'} + r_2 T_{L2} - \mu S_L + mp_{12}[S_H - S_L] \\
\frac{dD_L}{dt} &= \lambda\rho\Lambda_L S_L - aD_L + (\nu + d\Lambda_L)\rho A_L - \mu D_L + mp_{12}[D_H - D_L] \\
\frac{dA_L}{dt} &= (1-\lambda\rho)\Lambda_L S_L - gA_L - \sigma A_L - cA_L - (\nu + d\Lambda_L)\rho A_L - \mu A_L + mp_{12}[A_H - A_L] \\
\frac{dT_{L1}}{dt} &= aD_L - r_1 T_{L1} - \mu T_{L1} + mp_{12}[T_{H1} - T_{L1}] \\
\frac{dT_{L1}^{'}}{dt} &= r_1 T_{L1} - r_2 T_{L1}^{'} - \mu T_{L1}^{'} + mp_{12}[T_{H1}^{'} - T_{L1}^{'}] \\
\frac{dT_{L2}}{dt} &= cS_L - r_1 T_{L2} - \mu T_{L2} + mp_{12}[T_{H2} - T_{L2}] \\
\frac{dT_{L2}^{'}}{dt} &= r_1 T_{L2} - r_2 T_{L2}^{'} - \mu T_{L2}^{'} + mp_{12}[T_{H2}^{'} - T_{L2}^{'}] \\
\frac{dT_{L3}}{dt} &= cA_L - r_1 T_{L3} - \mu T_{L3} + mp_{12}[T_{H3} - T_{L3}], \\
\frac{dT_{L3}^{'}}{dt} &= r_1 T_{L3} - r_2 T_{L3}^{'} - \mu T_{L3}^{'} + mp_{12}[T_{H3}^{'} - T_{L3}^{'}] \\
\frac{dP_L}{dt} &= gA_L + r_2 T_{L1}^{'} + br_2 T_{L3}^{'} - \omega P_L - \gamma\Lambda_L P_L - \mu P_L + mp_{12}[P_H - P_L] \\
\frac{dS_{La}}{dt} &= \omega^{'} P_{La} - \Lambda_L S_{La} - \mu S_{La} + mp_{12}[S_{Ha} - S_{La}] \\
\frac{dD_{La}}{dt} &= \lambda^{'}\rho\Lambda_L S_{La} - aD_{La} + \nu^{'}\rho A_{La} - \mu D_{La} + mp_{12}[D_{Ha} - D_{La}] \\
\frac{dA_{La}}{dt} &= (1-\lambda^{'}\rho)\Lambda_L S_{La} - gA_{La} - \nu^{'}\rho A_{La} - \mu A_{La} + mp_{12}[A_{Ha} - A_{La}] \\
\frac{dT_{La}}{dt} &= aD_{La} - r_1 T_{La} - \mu T_{La} + mp_{12}[T_{Ha} - T_{La}] \\
\frac{dT_{La}^{'}}{dt} &= r_1 T_{La} - r_2 T_{La}^{'} - \mu T_{La}^{'} + mp_{12}[T_{Ha}^{'} - T_{La}] \\
\frac{dP_{La}}{dt} &= \gamma\Lambda_L P_L + r_2 T_{La}^{'} + gA_{La} - \omega^{'} P_{La} - \mu P_{La} + mp_{12}[P_{Ha} - P_{La}].
\end{aligned} \tag{1L}
$$

Models (1H) and (1L) form a 32×32 system of linear ordinary equations with one non-trivial steady state in 32 coordinates. The steady state value can be computed and its exact value numerically found for reasonable experimentally observed malaria related parameter ranges.

References

[1] O'Meara WP, Smith DL and McKenzie FE (2006). Potential impact of intermittent preventive treatment (IPT) on spread of drug resistant malaria. PLoS Medicine 3(5): 0633-0642.
