# Supplementary material for: The effect of intermittent preventive treatment on anti-malarial drug resistance spread in areas with population movement
Source: Malar J. 2014 Nov 15;13:428. doi: 10.1186/1475-2875-13-428 (PMC4289180; doi:10.1186/1475-2875-13-428)
Supplement: Supplementary file 2 — Additional file 2: This supplemental document contains supplemental figures showing results from the present spatial model for an average of a 10 days visitation when symmetric movement is considered. The results show that, overall, the qualitative results of the rate of spread of resistance with increases in IPT dosage is the same across a range of movement rate ratios in the low and high transmission areas. There is little distinction between an average of a 10 days visitation, a 50 days visitation, and a 100 days visitation (compare [Additional file 2: Figures S1, S2 and S3] to Figures 3, 6 and 10). The same is true whether considering the effects of IPT treatment with drugs with different half-lives or the effect of treatment on the rate of spread of resistance. Additional file 2 also contains graphical results that indicate the movement rates required to effect change in the non-spatial model in [18] (see Figures 5, 6 and 7) as well results indicating the profile of the SP drug compared to a drug with half-life that is four times that of the CPG-DDS drug (Figure 8). Moreover, Additional file 2: Table S3, which gives the parameters, their description, and values used in the model analysis. (DOCX 932 KB) [file 12936_2014_3653_MOESM2_ESM.docx]

Additional file 2

Figure S1: Effect of increasing IPT coverage with SP on the percent increase of R1 relative to RS and R2 relative to R1 resistance for p_12_ = 0.1 = p_21_ (10 days visitation), p_21_ / p_12_ = 1 = m. All other parameters are the same as those in the corresponding figures in the manuscript.

Figure S2: Effect of IPT treatment with different half-live drugs, SP or CPG-DDS, on the increase percentage of R1 relative to RS in both a low (Graph (a)) and high (Graph (b)) transmission setting, when symmetric movement between equally sized populations (p_12_=0.1 (10 days visitation), p_21_/p_12_=1=m) is considered. All other parameters are the same as those in the corresponding figures in the manuscript.

Figure S3: Effect of treatment on the rate of spread of resistance (in percent) when we consider symmetric movement between the high and low transmission areas so that m=1=p_21_/ p_12_, with p_21_=0.1 (10 days visitation). All other parameters are the same as those in the corresponding figures in the manuscript.

Figure S4: Effect of increasing IPT coverage with SP on the percent increase of R1 relative to RS and R2 relative to R1 resistance for p_12_ = 0.0001 = p_21_ (approx. a 27 years period), p_21_ / p_12_ = 1 = m. All other parameters are the same as those in the corresponding figures in the manuscript and given on Table 3 below. The graphs show that with symmetric movement, a movement rate of 0.0001 per day produced a qualitative change in the no movement result in relation to the speed of spread of R2 relative to R1 parasites in both the low and high transmission regions.

Figure S5: Effect of increasing IPT coverage with SP on the percent increase of R1 relative to RS and R2 relative to R1 resistance for p_12_ = 0.00015 = p_21_ (approx. an 18 years period), p_21_ / p_12_ = 1 = m. All other parameters are the same as those in the corresponding figures in the manuscript. The graphs show that with symmetric movement, a movement rate with a value slightly above 0.00015 per day is required to produce a qualitative change in the no movement result in both the low and high transmission regions.

Figure S6: Effect of increasing IPT coverage with SP on the percent increase of R1 relative to RS and R2 relative to R1 resistance for p_12_ = 0.0002 = p_21_ (approx. a 14 years period), p_21_ / p_12_ = 1 = m. All other parameters are the same as those in the corresponding figures in the manuscript. The graphs show that with symmetric movement, a movement rate of 0.0002 per day produced a qualitative change in the no movement result in both the low and high transmission regions.

Figure S7: Effect of IPT treatment with different half-live drugs, SP or a drug with half-life four times that of CPG-DDS, on the increase percentage of R1 relative to RS in both a low (Graph (a)) and high (Graph (b)) transmission setting, when symmetric movement between equally sized populations (p_12_=0.010 **(**100 days visitation), p_21_/p_12_=1=m) is considered. All other parameters are the same as those in the corresponding figures in the manuscript.

Table S3: Parameters, their description, their values. All values are taken from O’Meara et al. [18]

|  |  |  |  |
| --- | --- | --- | --- |
| Parameter | Description |  | Value used |
|  |  |  | in simulation |
|  |  |  |  |
| Λ | EIR: Infectious bites per person per day | In High | 1 per day |
|  |  |  |  |
|  |  |  |  |
|  |  | In Low | 0.01 per day |
|  |  |  |  |
| *λ* | Fraction of infected non immune individuals |  |  |
|  | who become symptomatic |  | 0.5 |
|  |  |  |  |
| *λ*^′^ | Fraction of infected semi-immune individuals |  |  |
|  | who become symptomatic |  | 0.25 |
|  |  |  |  |
| *c* | Number of IPTi treatments per person per day |  | 0.016 |
|  |  |  |  |
|  |  |  | 1*∕r*_1_=15 days |
|  |  |  |  |
|  |  |  |  |
| 1*∕r* | Time period of chemoprophylaxis | SP | 1*∕r*_2_=37 days |
|  |  |  |  |
|  |  |  |  |
|  |  |  | 1*∕r*_1_=1.2 days |
|  |  |  |  |
|  |  |  |  |
|  |  | CPG-DDS | 1*∕r*_2_=4.8 days |
|  |  |  |  |
| 1*∕a* | Number of days to clear infection via treatment |  | 5 days |
|  |  |  |  |
|  | Number of days to clear an infection via immune |  |  |
| 1*∕g* | mechanisms |  | 33 days |
|  |  |  |  |
|  | The fraction of asymptomatic treated individuals |  |  |
| *b* | immune protection |  | 0.5 |
|  |  |  |  |
|  | Length of time it takes for a non-immune |  |  |
| 1*∕w* | individual to stay temporarily immune |  | 28 days |
|  |  |  |  |
|  | Length of time it takes for a semi-immune |  |  |
| 1*∕w*^′^ | individual to stay temporarily immune |  | 370 days |
|  |  |  |  |
|  | The rate at which a temporary immune |  |  |
| *γ*Λ | non-immune transitions to a semi-immune state |  | 0*.*01Λ |
|  |  |  |  |
|  | Transition rate from asymptomatic infections to |  |  |
| *v* | symptomatic infections in non-immune | individuals | 0.01 |
|  |  |  |  |
|  | Transition rate from asymptomatic infections to |  |  |
| *v*^′^ | symptomatic infections in semi-immune | individuals | 0.05 |
|  |  |  |  |
|  | EIR-dependent transition rate from asymptomatic |  |  |
| *d* | to symptomatic infection |  | 0.1 |
|  |  |  |  |
| *ρ* | Probability that a symptomatic infection receives |  |  |
|  | treatment |  | 0.8 |
|  |  |  |  |
| *μ* | Birth and death rate |  | 5*.*5 × 10^-5^ |
|  |  |  | per day |
|  |  |  |  |
|  | Fraction of infected individuals who can be |  |  |
| *q* | superinfected (infected more than once) |  | 0.1 |
|  |  |  |  |
|  | Rate at which an infection is cleared with no |  |  |
| *σ* | immunity development |  | 0.01 |
|  |  |  |  |
|  | Ratio of the total humans in the high transmission |  | varies: |
| *m* | area to that of the low transmission (*N_H_∕N_L_*) |  | 1, 2, 1/2 |
|  |  |  |  |
|  | Time a high transmission human resident |  |  |
| 1*∕p*_12_ | spends visiting the low transmission area |  | varies |
|  |  |  |  |
|  | Time a low transmission human resident |  |  |
| 1*∕p*_21_ | spends visiting the high transmission area |  | varies |
|  |  |  |  |
|  |  |  |  |
